# Skin Sensitization Potential of Sensitizers in the Presence of Metal Oxide Nanoparticles In Vitro

**DOI:** 10.3390/nano14221811

**Published:** 2024-11-12

**Authors:** Claudia Meindl, Kristin Öhlinger, Verena Zrim, Jennifer Ober, Ramona Jeitler, Eva Roblegg, Eleonore Fröhlich

**Affiliations:** 1 Center for Medical Research, Medical University of Graz, Stiftingtalstr. 24, 8010 Graz, Austria; claudia.meindl@medunigraz.at (C.M.); kristin.oehlinger@medunigraz.at (K.Ö.); verena.zrim@medunigraz.at (V.Z.); jennifer.ober@medunigraz.at (J.O.); 2 Department of Pharmaceutical Technology, Institute of Pharmaceutical Sciences, Karl-Franzens-University of Graz, Humboldtstr, 46, 8010 Graz, Austria; ramona.jeitler@uni-graz.at (R.J.); eva.roblegg@uni-graz.at (E.R.)

**Keywords:** metal oxide nanoparticles, sensitization, skin, in vitro assay, toxicity, titanium dioxide, silica, zinc oxide

## Abstract

Silica (SiO_2_), titanium dioxide (TiO_2_), and zinc oxide (ZnO) nanoparticles (NPs) are widely used in dermal products. Their skin sensitization potential, especially their effects in combination with known sensitizers, is poorly studied in vitro and their sensitization inconsistently reported in animal studies. In this study, cellular assays were used to identify different steps of sensitization, the activation of keratinocytes and dendritic cells, when cells were exposed to these NPs in the absence and presence of sensitizers. Cellular systems included HaCaT keratinocytes and U937 (U-SENS™) alone, as well as different co-culture systems of THP-1 cells with HaCaT cells (COCAT) and with primary keratinocytes. The effect of NPs differed between co-cultures and U-SENS™, whereas co-cultures with either primary keratinocytes or HaCaT cells responded similarly. Pre-exposure to ZnO NPs increased the U-SENS™ assay response to 2,4-dinitrochlorobenzene six-fold. The COCAT increase was maximally four-fold for the combination of SiO_2_ and trans cinnamaldehyde. When the THP-1 cells were separated from the keratinocytes by a membrane, the response of the co-culture system was more similar to U-SENS™. The direct contact with keratinocytes decreased the modulating effect of TiO_2_ and ZnO NPs but suggested an increase in response to sensitizers following dermal contact with SiO_2_ NPs.

## 1. Introduction

Silica (SiO_2_), titanium dioxide (TiO_2_), and zinc oxide (ZnO) nanoparticles (NPs) constitute a triad of the most commonly utilized oxide NPs. Humans are exposed to SiO_2_ particles in a number of ways, including in the workplace, through the consumption of food additives and dietary supplements, in the use of cosmetic products, and in medicine [1]. TiO_2_ NPs have been extensively produced as whitening, anti-caking, and coloring agents in a variety of products, including paints, cosmetics, and foodstuffs [2]. Over 70% of all ZnO NPs are utilized in personal care products, including sunscreens, and they also serve a role in antibacterial food packaging materials [3]. TiO_2_ and ZnO (micro)-particles serve as the foundation for a range of anti-inflammatory and antifungal dermatological formulations [4].

One of the principal routes of exposure for these NPs is dermal exposure. SiO_2_ nanoparticles (5–100 nm) as agents for thickening, abrasion, anti-caking, mattifying, and volumizing are used in personal care products [5]. TiO_2_ nanoparticles are 10–150 nm and ZnO NPs 8–64 nm in cosmetics [6]. TiO_2_ and ZnO NPs are mainly used in sun protection, and SiO_2_ nanoparticles in temporary hair styling, lipstick, toothpaste, eyeliner, and eye shadow [7].

Cytotoxicity, irritation, and sensitization tests are considered the primary tests for assessing biocompatibility [8]. Skin sensitization is a common toxicity endpoint of concern and accounts for 10–15% of known occupational illnesses in the U.S. and Europe [9]. There is a greater abundance of data pertaining to the cytotoxicity of these NPs in comparison to their potential as sensitizers. One potential explanation for this discrepancy is that regulatory tests for skin irritation (OECD 439) and skin corrosion (OECD 431) can be conducted using reconstructed human epidermis, whereas regulatory authorities only accept animal tests for sensitization assessment. Accordingly, the local lymph node assay (LLNA) has emerged as the gold standard for skin sensitization testing (OECD 442A: Skin Sensitization) [10]. The proliferation observed in the LLNA can be quantified through the incorporation of ^3^H-thymidine (OECD 442A) or by utilizing flow cytometry with BrdU (OECD 442B).

At present, there are no approved alternatives to the established in vivo tests, in part because the process of skin sensitization, a type IV hypersensitivity, is a complex one that comprises four distinct phases: (i) interaction with skin proteins to form the hapten-protein complex, (ii) the inflammatory response of keratinocytes, (iii) the activation of dendritic cells (DCs), and (iv) the proliferation and activation of T cells [11]. The mouse LLNA is the sole test that assesses all phases, whereas the guinea pig maximization test and the Buehler test evaluate only phases I–III.

The sensitizing potential of the three NPs under investigation has been the subject of considerable debate in the literature, with reports from in vivo experiments indicating both positive and negative outcomes. SiO_2_ NPs (10–20 nm) did not elicit an immune response in the LLNA assay [12]. In another publication, 20 nm SiO_2_ NPs were observed to increase skin thickness when injected intradermally, while other parameters such as the degranulation of basophils and secretion of cytokines were not affected by the NPs [13]. It is crucial to examine the response when the NPs are combined with sensitizers, as 14 nm SiO_2_ and 20–80 nm TiO_2_ have been shown to activate co-stimulatory molecules (CD80, CD86, MHC II) in murine DCs [14]. The administration of 30 to 100 nm SiO_2_ NPs resulted in the exacerbation of atopic dermatitis induced by *Dermatophagoides pteronyssinus*. Larger particles (300–1000 nm) demonstrated smaller effects [15]. The study did not provide any information regarding the effect of NPs in the absence of a sensitizer.

The topical application of 15 nm TiO_2_, but not 19 nm SiO_2_ NPs, was observed to enhance the response to 2,4-dinitrochlorobenzene (DNCB) in the LLNA, whereas the administration of both NPs individually did not elicit any discernible effect [16]. Similarly, the subcutaneous injection of 15 nm TiO_2_ NPs prior to topical contact with the skin sensitizer DNCB was observed to increase Interleukin (IL)-4 production and cell proliferation in the lymph node [17]. The particles exhibited no evidence of sensitization when administered alone. The absence of a difference between the topical and injected NPs lends support to the concept that activated DCs are capable of taking up antigens outside the skin barrier [18]. However, another study reported a discrepancy between the effects of topical administration and subcutaneous injection [19]. TiO_2_ NPs of <25 nm were observed to induce LLNA exclusively following subcutaneous administration. This result may be explained by the fact that the uppermost layer of the epidermis, the stratum corneum, represents the epithelial barrier of the skin, and intradermal injection circumvents this barrier [20]. One study indicated that ZnO NPs with a diameter of 20 nm and 240 nm led to a reduction in skin thickness, cell infiltration, and cytokine secretion in an atopic dermatitis mouse model [21]. A single exposure to the NPs demonstrated a reduction in cytokine expression in skin biopsies. This effect differs from that observed in the guinea pig sensitization assay, in which 396 nm ZnO NPs were classified as mild sensitizers [22]. In conclusion, no effect or stimulation was observed for 10–20 nm SiO_2_ and 15–25 nm TiO_2_ NPs, while both suppressive and stimulatory effects were reported for 20–240 nm ZnO. In combination with sensitizers, 20–100 nm SiO_2_ has the potential to elicit a range of effects, including suppression, neutrality, and stimulation. Similarly, 15 nm TiO_2_ has been observed to elicit stimulation, while 20–240 nm ZnO NPs have been shown to suppress effects. These disparate outcomes have been documented for NPs within a comparable size range, suggesting that exposure parameters and animal models exert a considerable influence on the results. As a result, the interpretation of the results is challenging.

In accordance with Regulation EC No 1223/2009 of the European Parliament and of the Council of 20 November 2009 on cosmetic products, the use of animals for testing the final formulation, ingredients, or raw materials of cosmetic products is prohibited [23]. This legislation is being enacted concurrently with similar restrictions in other countries. These regulations, in conjunction with the reinforcement of the replacement, reduction, and refinement (3Rs) of animal testing, are stimulating the use and development of cellular assays. Cellular models are unable to reflect the complexity of the organism, but they do possess the advantage of allowing the assessment of different steps of the sensitization process. The THP-1 and U973 cells are well-established surrogate models for DCs and are employed in the h-CLAT (THP-1) and U-SENS™ assays [24]. HaCaT cells are regarded as the most suitable model for studying keratinocytes [25]. To compare the response of NPs alone and in the presence of sensitizers, three standard NPs (TiO_2_, SiO_2_, and ZnO) obtained from the Joint Research Centre (JRC) of the European Commission were subjected to analysis. The selection of these particles was based on the observation that they are the most commonly used nanoparticles in cosmetics and sun protection. As these products are used by the majority of the population, the potential for skin sensitization is a significant concern. The strong sensitizer DNCB, and the moderate sensitizer trans cinnamaldehyde (TCA), were employed for the activation of DCs [26] and DNCB for the co-culture systems [27].

The strategy of sensitization testing in vitro is based on the use of different assay systems, including the direct peptide reactivity assay (OECD 442C), the keratinocyte activation assay (OECD 442D), and the activation of DC assay (OECD 442E). The classification of NPs as sensitizers based on the results of in vitro assays is a challenging process. In general, a substance is classified as a sensitizer if it reacts positively in two out of the three in vitro assays. In a study by Wareing et al., a panel of NPs was tested, and the results of the direct peptide reactivity assay were found to be inconclusive [28]. Moreover, the outcomes of the h-CLAT assay for DC activation were inclusive for various SiO_2_ NPs, including those of varying sizes (5–50 nm, 9 nm, and 16 nm). The 190 nm ZnO NPs, which had been identified as a sensitizer in vitro, did not act as a sensitizer in the human patch test. This prompts the question of whether exposure to a single cell type is sufficient for the classification.

In this study, the NPs are exposed to mono- and to co-cultures of keratinocytes and antigen-presenting cells to evaluate the importance of direct interaction between these cells. The cellular systems that were studied included a monoculture of HaCaT cells, a monoculture of U937 monocytes (U-SENS™), a co-culture of HaCaT keratinocytes and THP-1 monocytes (Cocultured Activation Test, COCAT), a co-culture of primary keratinocytes and THP-1 monocytes (COCAT_primary keratinocytes), and HaCaT on membranes and THP-1 monocytes (COCAT_transwell).

## 2. Materials and Methods

### 2.1. Cells

Human epidermal keratinocytes HaCaT were obtained from the American Type Culture Collection (ATCC, Manassas, VA, USA) and cultured in Dulbecco’s modified Eagle’s medium (DMEM) supplemented with 10% fetal bovine serum (FBS), 1% streptomycin/penicillin in an atmosphere of 5% CO_2_ at 37 °C. Human THP-1 monocytes were purchased from Cell Line Services (Eppelheim, Germany) and cultured in Roswell Park Memorial Institute medium (RPMI)-1640 supplemented with 2 mM L-glutamine, 1% penicillin/streptomycin, and 10% FBS. Human U937 histiocytic lymphoma cells were obtained from ATCC and cultured in RPMI-1640 supplemented with 2 mM L-glutamine, 1% penicillin/streptomycin, and 10% heat-inactivated FBS.

Primary keratinocytes isolated from human foreskin were obtained from the cell bank of the Medical University of Graz (Graz, Austria). Keratinocytes were cultured in keratinocyte growth medium (Epilife with calcium) supplemented with Human Keratinocyte Growth Supplement 100× (S0015, Life Tech, Carlsbad, CA, USA) to a final concentration of 0.2% *v*/*v* bovine pituitary extract, 0.2 ng/mL human epidermal growth factor, 0.18 µg/mL hydrocortisone, 5 µg/mL bovine transferrin, and 0.01 µg/mL human recombinant insulin-like growth factor. The medium was changed every two days and cells were used for experiments at passage 4–6.

### 2.2. Chemicals

Picrylsulfonic acid (2,4,6,-Trinitro-benzene-sulfonic acid, TNBS), trans cinnamaldehyde (TCA), and 2,4-dinitrochlorobenzene (DNCB) were obtained from Sigma Aldrich, Vienna, Austria. Furthermore, isopropranol (2-propanol, Merck, Vienna, Austria), lactic acid (LA, VWR, Vienna, Austria), paraformaldehyde (PFA, Gatt-Koller, Absam, Austria), and sodium dodecyl sulfate (SDS, Roth, Karlsruhe, Germany) were used.

### 2.3. Characterization of NPs

Particles were obtained from the Joint Research Center of the European Commission (JRC, Ispra, Italy) and consisted of 14–23 nm precipitated amorphous SiO_2_ (JRCNM02000a), 5–6 nm anatase TiO_2_ (JRCNM01001a), and 70–90 nm ZnO (JRCNM62101a) particles (see Table S1 Additional particle information).

The hydrodynamic sizes (Z-Ave; nm) and the polydispersity index (PdI) of SiO_2_, TiO_2_, and ZnO particles in the exposure media were determined via Dynamic Light Scattering (DLS) using the Zetasizer Nano ZS (Malvern Instruments Ltd., Malvern, UK, Zetasizer Software Version 8.02). Particle suspensions were prepared at a concentration of 2.5 mg/mL in RPMI-1640 + 10% FBS (U-SENS™) or in DMEM + 10% FBS (MTS, interleukin secretion, COCAT) and were treated with ultrasound (Elmasonic S40 water bath with ultrasonic frequency: 37 kHz, Elma Schmidbauer GmbH, Singen, Germany) for 20 min. Prior to measurements, the dispersions were diluted with the respective exposure media to avoid multiple scattering and hindered diffusion. Measurements were performed in triplicate at 37 °C using a measurement angle of 173°. The zetapotential of the NPs dispersed in the exposure media was measured via Electrophoretic Light Scattering using the same equipment and measurement angle. To evaluate stability of the suspensions, measurements were repeated in the same manner after 24 h. Each measurement was conducted three times.

### 2.4. Viability Assay (MTS)

HaCaT cells were seeded in subconfluent condition in 96-well plates 24 h before exposure to compounds and NPs. The CellTiter 96^®^ Aqueous non-radioactive cell proliferation assay (Promega, Mannheim, Germany) was used. For NPs, the highest particle concentration in the absence of cells (interference control) and for chemicals, the highest concentration of organic solvent (solvent control) were included in the design. Triton X-100 (0.1%) was used as a positive (toxic) control. The assay was performed according to the protocol provided by the manufacturer. Briefly, 20 µL of the combined MTS/PMS solution + 100 µL medium were added to each well. Plates were incubated for up to 2 h at 37 °C in a cell incubator. Absorbance was read at 490 nm on a plate reader (SPECTRA MAX plus 384, Molecular Devices, Wokingham, UK).

### 2.5. Interleukin (IL)-6 and IL-1α Detection

HaCaT cells were seeded in 24-well plates and treated with LA, isopropranol, PFA, TCA, and 2,4-DNCB at non-cytotoxic concentrations. After 24 h of exposure, supernatants were harvested and cytokine levels determined using DuoSet ELISA kit (Bio-Techne GmbH, Wiesbaden, Germany) for IL-1α and IL-6 OptEIA Elisa Set (BD Biosciences, Vienna, Austria). Absorbance was read at 450 nm (with correction wavelength of 570 nm) on a SPECTROstar (BMG Labtech, Ortenberg, Germany) photometer.

### 2.6. U-SENS™ Assay

The assay was performed according to the OECD 442E guideline [29] using an increase in CD86 expression >150% or CD54 expression >200% as the cut-off for sensitizer identification [30]. U937 cells (passages 9–17) were seeded at a final cell density of 0.5 × 10^5^ cells/well in a 96-well plate. Test compounds were added for 45 h in RPMI + 10% FBS. TNBS was used as a positive control and LA as a negative control. PFA, TCA, and DNCB were used as additional sensitizers. The concentrations were chosen so that the viability of the cells was >70% at the end of the exposure. Chemicals (DNCB, TCA) were freshly dissolved in dimethyl sulfoxide (DMSO, final concentration 0.2%) or cell culture medium. Freshly prepared NP suspensions were added and incubated in the same manner as the conventional substances.

For co-exposures, NPs were either added together with the sensitizers for the entire duration of exposure or NPs were added only for the last 24 h of exposure to the sensitizer.

After the exposure, cells were collected by centrifugation, washed once with phosphate buffered saline (PBS) containing 3% FBS, and stained with FITC-labelled anti-CD86 antibody (1:10, BD Biosciences, BD Austria GmbH, Vienna, Austria) in PBS containing 3% FBS for 20 min in the dark at room temperature. Cells were washed once with PBS containing 3% FBS and re-suspended in ice-cold PBS containing 3% FBS and propidium iodide (PI, BD Biosciences, BD Austria GmbH, Vienna, Austria) at a final concentration of 0.75 µg/mL. Cells were analyzed using CytoFLEX S (Beckman Coulter, Vienna, Austria) and CytExpert software version 2.6 (Beckman Coulter).

The stimulation index (S.I.) was calculated as:

$$S.I.=\frac{\%\;\mathrm{of}\;\mathrm{CD}86\mathrm{pos}\;\mathrm{treated}\;\mathrm{cells}\;-\;\%\;\mathrm{of}\;\mathrm{IgG}\;\mathrm{treated}\;\mathrm{cells}}{\%\;\mathrm{of}\;\mathrm{CD}86\mathrm{pos}\;\mathrm{control}\;\mathrm{cells}\;-\;\%\;\mathrm{of}\;\mathrm{IgG}\;\mathrm{control}\;\mathrm{cells}}$$

A substance is classified as a sensitizer if the stimulation index (S.I.) is ≥150% and viability ≥ 70% according to PI exclusion. Control cells were treated with solvent (DNCB, SDS) or medium (TCA, NPs). In the co-exposures with the NPs, the % of CD86pos cells was related to the respective sensitizer or negative control.

### 2.7. Co-Cultured Activation Test (COCAT)

**Figure 1:**
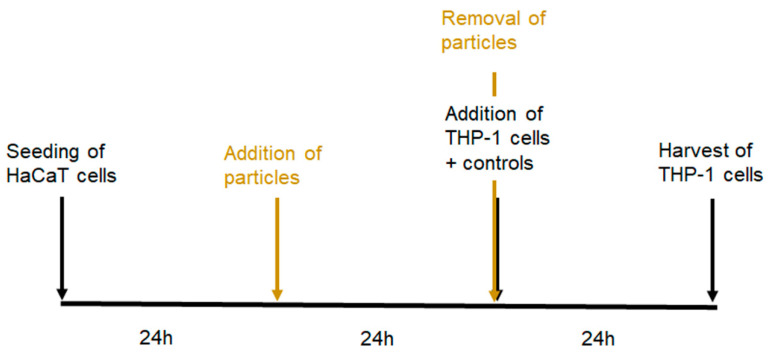
Layout of the COCAT. Workflow of the routine assay is indicated in black, and adaptations for the co-exposure to NPs are indicated in brown.

COCAT_HaCaT is a co-culture of HaCaT keratinocytes and THP-1 cells, and was found promising for identifying skin sensitizers. The optimized Standard Operating Protocol (SOP) used in this study was published by Eskes et al. [27] and the treatment scheme is illustrated in Figure 1. For the combined treatment with NPs, the protocol was adapted in the way that HaCaT cells were exposed to the particles alone for 24 h prior to removal of the particles and the addition of THP-1 cells and chemicals.

On the first day, 2.5 × 10^4^ HaCaT cells were seeded in 200 µL DMEM + 10% FBS per well of a 96-well plate and cultured for 48 h. Particles suspended in DMEM + 10% FBS were added after 24 h. On day three, medium was removed and 8 × 10^4^ THP-1 cells plus controls in 180 µL RPMI medium + 10% FBS and Hepes were added. THP-1 cells were initially used at low passage numbers (8–18) and high passage numbers (50–58). THP-1 at higher passages showed variable cytotoxicity and only THP-1 cells at low passage numbers were used in the final experiments.

Test substances’ final concentrations of positive controls: 3 µg/mL DNCB and 1.5 µg/mL TCA were freshly dissolved in dimethyl sulfoxide (DMSO, final concentration of 0.2%). The negative control (41.5 µg/mL SDS) and NPs were applied in RPMI + 10% FBS medium. After 24 h treatment, the floating THP-1 cells were harvested and washed with PBS. For the staining with anti-human antibodies, three wells were pooled and stained with FITC-labelled anti-CD86 (1:20; BD Biosciences, #555657) and APC-labelled anti-CD54 antibodies (1:20; Beckman Coulter, #IM1239U) for 20 min in the dark at room temperature. The viability of THP-1 cells was determined by PI (BD Biosciences, #556463). CD86 and CD54 expression was analyzed using CytoFLEX S (Beckman Coulter, Vienna, Austria) with CytExpert software.

Similar to the U-SENS™ assay, we used the S.I. > 150% in CD86 expression as an indicator for sensitizing agents. In the co-exposures with the NPs, the % of CD86pos cells was related to the respective sensitizer or negative control. For DNCB and TCA, solvent-treated cells served as controls and for SDS and NP exposures the medium RPMI. Only experiments where all exposures showed cell viability >50% were included.

In the COCAT_primary keratinocytes assay, 3.5 × 10^4^ primary keratinocytes were seeded per well of a 96-well plate, and the same protocol was followed as for the COCAT assay.

### 2.8. COCAT_Transwell Culture

This culture was used to mimic the dermal barrier where the stimulants first come into contact with the keratinocytes. In pilot experiments, the co-culture and the addition of the stimulants were established. It was found that the response to the sensitizers was very low when they were added to the HaCaT cells in the apical compartment and the THP-1 cells were cultured in the basal compartment, and the following protocol was used.

Two × 10^5^ HaCaT cells were seeded per 12-well transwell insert (Greiner Bio-One, Kremsmünster, Austria, 0.4 µm pore size) in 500 µL DMEM + 10% FBS in the apical compartment and 1500 µL of the same medium in the basal compartment. After six days, the cells had formed layers consisting of two rows of cells. SiO_2_ (12.5; 25 µg/mL), TiO_2_ (12.5; 25 µg/mL), and ZnO (6.25; 12.5 µg/mL) NPs dispersed in 500 µL DMEM + 10% FBS were added to the HaCaT cells in the apical compartment. After 24 h, 2.5 × 10^5^ THP-1 cells in RPMI + 10% FBS were added to the apical compartment and 3 µg/mL DNCB to the basal compartment. After another 24 h, THP-1 cells were harvested and washed three times with PBS. The analysis of CD86 and CD54 expression was performed as described for COCAT exposure.

### 2.9. Statistics

Experiments in 2–3 repetitions with duplicates or triplicates per repetition were performed and mean ± SD were indicated. S.I. > 150% of CD86 expression was used for the classification of substances or NPs as sensitizer. Changes in the activation index by exposure with the NPs were analysed after applying Levene’s Test for Equality with independent sample *t*-test (IBM SPSS Statistics 28, Vienna, Austria), and a *p*-value of <0.05 was regarded as significant.

## 3. Results

### 3.1. Characterization of NPs

The hydrodynamic diameters of the nanoparticles (NPs) in fetal bovine serum (FBS)-containing media were significantly larger than their primary sizes (Table 1). The polydispersity indices (PDIs) were found to exceed 0.3 for all suspensions. In accordance with the definition of nanocarrier systems, this signifies a broad distribution [31]. The low zeta potential observed for all particles indicates that the suspensions are unstable. The changes in hydrodynamic size, polydispersity index (PDI), and zeta potential were systematically investigated in different suspension media for 22 nm SiO_2_ NPs [32]. The authors observed the most pronounced increases in hydrodynamic sizes in FBS-containing media at elevated particle concentrations for freshly prepared dispersions. The observed increase in particle size was attributed to the agglomeration of primary nanoparticles. Similar changes were also seen in our suspensions—there were no marked differences between the hydrodynamic sizes of particles dispersed in RPMI + 10% FBS and in DMEM + 10% FBS, and the relative size increase was greater for the smaller (SiO_2_ and TiO_2_) than for the larger ZnO nanoparticles. As evidenced by the high PDI values, the suspensions exhibited a high degree of polydispersity. The diminished average sizes observed after 24 h of incubation indicate that only a few larger agglomerates were formed, which did not contribute to the average size. These data indicate that the cells interact primarily with the NPs as agglomerates, and that the agglomeration process continued during the incubation period.

### 3.2. Cell Culture Models and Detection Method

**Figure 2:**
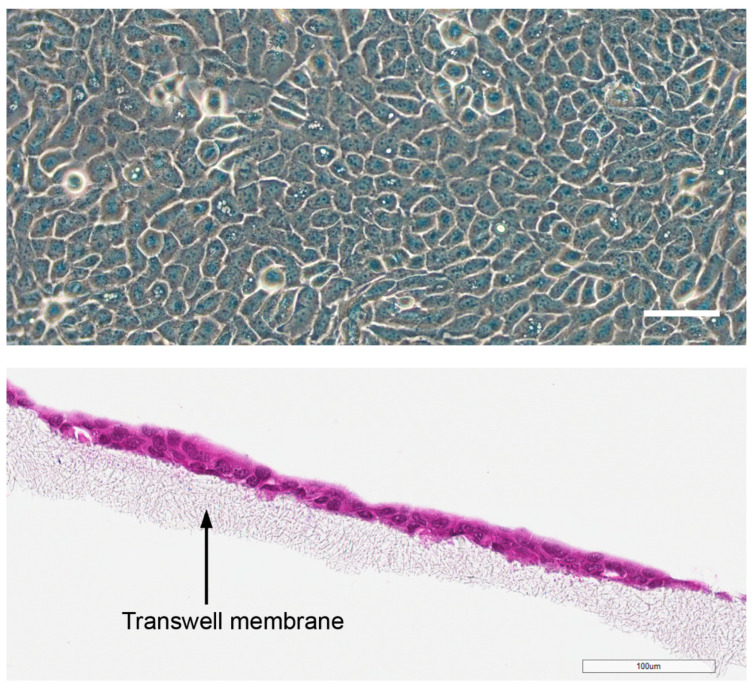
HaCaT cells cultured in plastic wells (phase contrast) and on transwell membrane inserts (stained with HE). The cobblestone morphology is typical for the keratinocytes in plastic wells, and HaCaT cells form a stratified epithelium on membranes, similar to keratinocytes in normal skin. Scale bar: 100 µm.

HaCaT cells were exposed in the conventional COCAT in plastic wells at confluence (Figure 2). In the COCAT_transwell, where the cells were cultured on Transwell membranes, a stratified layer (two rows of cells) was formed.

**Figure 3:**
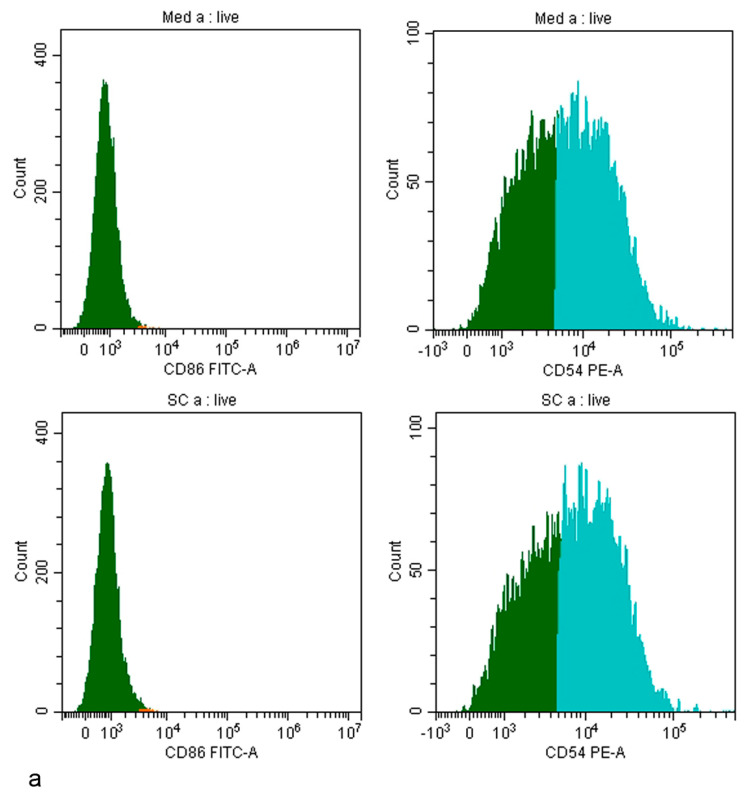

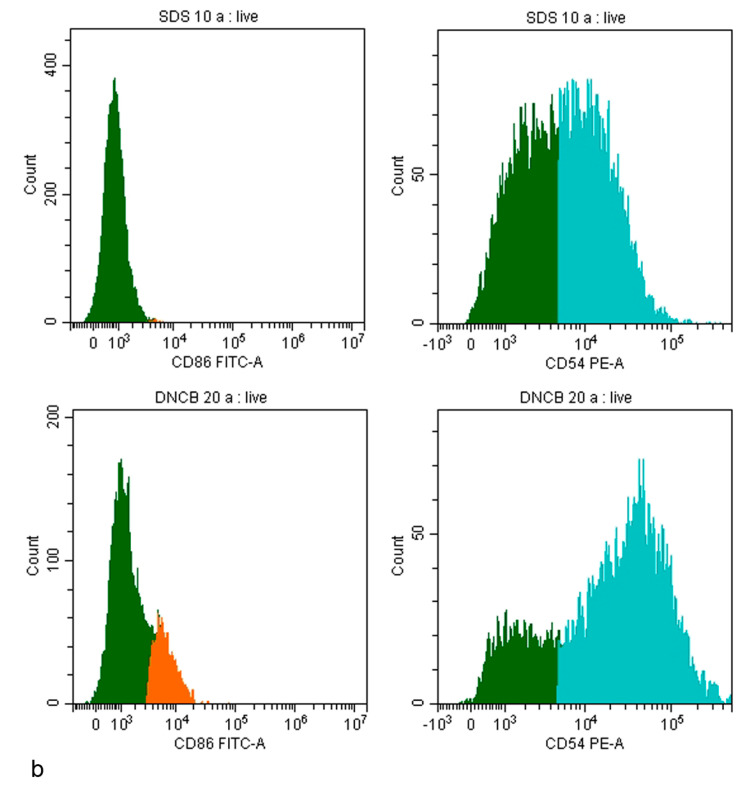
Histograms of THP-1 cells stained with CD86 FITC and CD54 PE antibody. Cells gated based on the unstained controls are indicated for CD86 in red and for CD54 in green. Histograms of growth control (med) and solvent control (SC) (**a**), negative control (SDS) and positive control (DNCB) (**b**), are shown.

The analysis of CD expression was performed by flow cytometry. Typical histograms of media (Figure 3a), and positive and negative controls (Figure 3b), are shown.

### 3.3. Compound and Particle Cytotoxicity

Cytotoxicity was determined for monocytes in each experiment by flow cytometry. Solely experiments in which the required viability was reached in all samples and controls were used for the analysis. For the U-Sens™ assay, viability was required to exceed 70%, while for the COCAT assays, viability had to exceed 50%.

**Figure 4:**
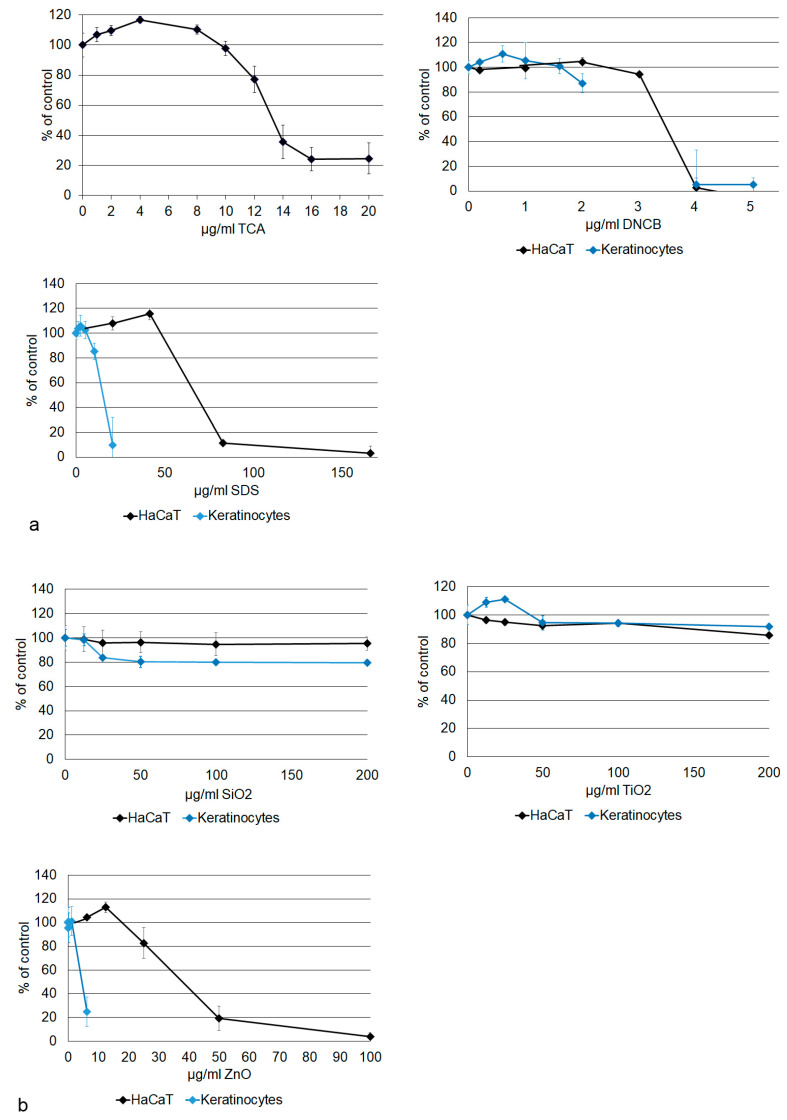
Cell viability is indicated as % of (medium or solvent) control. Viability was determined for trans cinnamaldehyde (TCA) in HaCaT keratinocytes (**a**). 2,4-dinitrochlorobenzene (DNCB), sodium dodecylsulfate (SDS) (**a**), SiO_2_, TiO_2_, and ZnO (**b**), were tested in HaCaT and primary keratinocytes.

In the co-exposure experiments utilizing keratinocytes (HaCaT and primary cells), cytotoxicity was assessed through the measurement of dehydrogenase activity, as determined by the MTS assay. Determination of keratinocyte viability is important because cytotoxic effects may influence the activation of THP-1 cells. HaCaT cells in conventional culture demonstrated cytotoxicity to the sensitizers TCA at concentrations exceeding 10 µg/mL, DNCB at concentrations exceeding 3.03 µg/mL (Figure 4a), and PFA at concentrations exceeding 4 µg/mL (Figure S1, Supplementary Material). The negative control SDS was not observed to exert any cytotoxic effects at concentrations of ≤41.5 µg/mL (Figure 4a), isopropranol at ≤15 mg/ml and LA at the highest concentration tested (≤1.4 mg/mL) (Figure S1, Supplementary Material). NP concentrations of ≤200 µg/mL SiO_2_ and TiO_2_, and of ≤50 µg/mL ZnO, did not result in a viability reduction to less than 70% of the growth controls (Figure 4b).

The response of primary keratinocytes to DNCB, SiO_2_, and TiO_2_ was similar, however, primary cells were significantly more sensitive to SDS (IC50 = 57.3 ± 0.7 µg/mL for HaCaT and IC50 = 13.6 ± 0.3 µg/mL for primary keratinocytes) and ZnO (IC50 = 33.9 ± 0.1 µg/mL for HaCaT and IC50 = 8.7 ± 0.0 µg/mL for primary keratinocytes).

In the final experiments, DNCB and TCA were employed as sensitizers, while SDS served as a non-sensitizer in the COCAT assay. In the COCAT_primary keratinocyte and COCAT_transwell assays, DNCB was utilized as a sensitizer, with SDS acting as a non-sensitizer.

### 3.4. Interleukin Secretion by HaCaT Cells as an Indicator of Sensitization

Secretions of IL-6 and IL-1α have been demonstrated to be suitable for differentiating sensitizers from non-sensitizers [33]. These cytokines were determined upon exposure to TCA (0.12–13 µg/mL), DNCB (0.008–0.6 µg/mL), and isopropanol (0.15–4 mg/mL) as sensitizers and LA (0.013–1.3 mg/mL) as non-sensitizers, within the respective non-cytotoxic range. The IL-6 levels exhibited a positive response (3.2–16.16 pg/mL) upon exposure to 0.01–0.30 µg/mL DNCB, whereas all other conditions yielded negative results. However, the response was not dose-dependent and exhibited inconsistency. Moreover, no IL-1α secretion was observed in any of the samples. Accordingly, this assay was deemed unsuitable for the assessment of NP effects on sensitization.

### 3.5. U-Sens™ Assay

To obtain the highest increase in CD86 expression, 1–200 µg/mL TNBS, 0.25–1 µg/mL TCA, 0.05–2 µg/mL PFA, and 1–400 µg/mL LA were evaluated. According to the guideline (viability > 70% and S.I. > 150%), 25 µg/mL TNBS, 1 µg/mL TCA, 0.4 µg/mL PFA, and 0.5 µg/mL DNCB were identified as sensitizers (Table 2). The S.I. at all concentrations of LA was <150%, and 50 µg/mL LA was used as a negative control.

NPs were evaluated in a concentration range of 12.5–200 µg/mL TiO_2_, 3.12–200 µg/mL SiO_2_, and 3.12–50 µg/mL ZnO. U937 showed >90% viability when exposed to TiO_2_ up to 200 µg/mL. Viability of >70% was also observed for concentrations of 3.12–50 µg/mL SiO_2_ and 3.12–25 µg/mL ZnO. None of the particles had a sensitizing effect (Table 2) as the S.I. was <150%.

**Figure 5:**
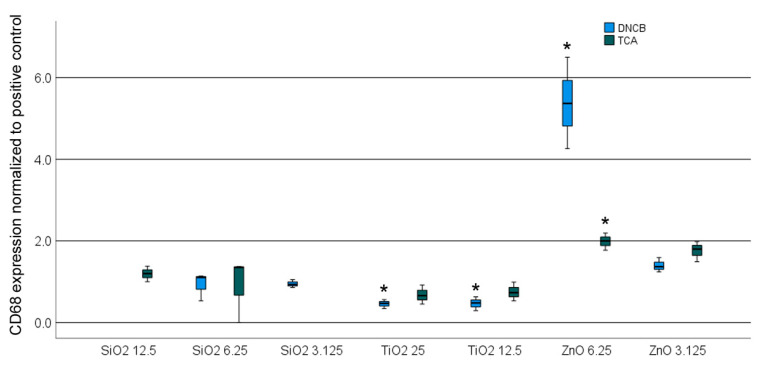
Expression of CD86 by U937 cells in the U-Sens™ Assay after exposure to the NPs in combination with 2,4-dinitrochlorobenzene (DNCB) and trans cinnamaldehyde (TCA) as positive control (PC). The expression is normalized to CD86 expression induced by the sensitizer alone. Significant changes (*p* < 0.05) are indicated by an asterisk.

To avoid the direct binding of the chemicals to the NPs, the particles were introduced solely during the final 24 h of exposure. Co-exposure with TCA resulted in a reduction in the non-cytotoxic concentration of SiO_2_ from 200 µg/mL to 50 µg/mL, and that of ZnO from 25 µg/mL to 12.5 µg/mL. To exclude any cytotoxic effects, TiO_2_ and SiO_2_ were utilized at concentrations of 25 µg/mL and 12.5 µg/mL, respectively, while ZnO was employed at concentrations of 6.25 µg/mL and 3.125 µg/mL. The effect of TCA and DNCB on NPs at non-cytotoxic concentrations was not significantly dose-dependent, with the exception of ZnO in combination with DNCB (Figure 5). SiO_2_ did not affect the response to TCA and DNCB, whereas exposure to ZnO increased, and that to TiO_2_ decreased, the response to the sensitizers significantly.

### 3.6. COCAT Assay

The surface expression of CD54 and CD86 on THP-1 cells was determined in the absence and presence of HaCaT cells. The presence of HaCaT cells resulted in no marked increase in CD54 expression of THP-1 cells from 62 ± 2.3% to 63 ± 1.0% in the medium, and from 59 ± 2.1% to 68 ± 1.0% in the solvent DMSO. The expression of CD86 remained unchanged at 0.7% in the medium, and 0.5% and 0.6% in the solvent. However, THP-1 cells exhibited heightened sensitivity to 3 µg/mL DNCB as a positive control, with an S.I. of 128 ± 5 in the presence and 89 ± 10 in the absence of HaCaT cells for CD54, and an S.I. of 2786 ± 638 in the presence and 355 ± 38 in the absence of HaCaT cells for CD86.

To simulate the combined treatment of chemicals and NPs, the most realistic exposure scenario was estimated to be the pretreatment of HaCaT keratinocytes with NPs followed by particle removal. This approach prevents the direct stimulation of THP-1 cells by NPs, which reflects the situation in vivo, where limited skin penetration results in particle contact primarily with keratinocytes [34]. While the known sensitizers TCA and DNCB were observed to increase CD54 and CD86 expression in co-culture with HaCaT cells, the negative control SDS did not result in any increase in CD marker expression (Table 3). With the exception of SiO_2_ for CD86 expression, no stimulation was observed for the NPs.

**Figure 6:**
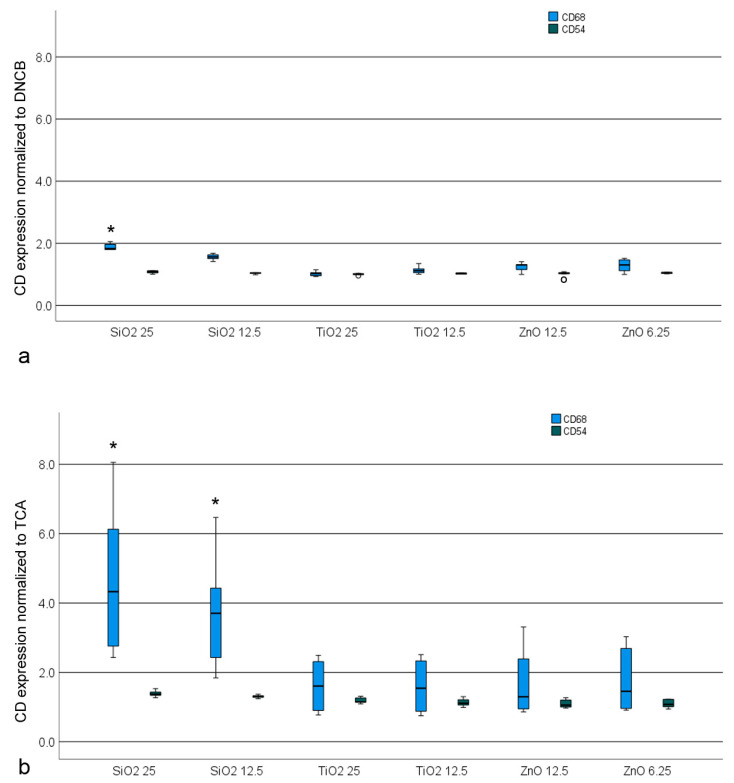
CD86 and CD54 expression of THP-1 cells when HaCaT cells were exposed to NPs prior to contact with the positive controls (PCs), 2,4-dinitrochlorobenzene (DNCB, (**a**)) and trans cinnamaldehyde (TCA, (**b**)). Significant changes (*p* < 0.05) are indicated by an asterisk.

Following the pre-exposure of HaCaT to NPs, the response of CD86 expression to TCA was enhanced for all NPs, although this response was only statistically significant for SiO_2_ exposure (Figure 6). The basal response of the co-culture to DNCB was greater than that to TCA, however, the increase in response by NPs was more pronounced for TCA. Previous exposure to SiO_2_ particles exerted a significant influence on the response to DNCB. The NPs did not induce notable alterations in CD54 expression elicited by the sensitizers.

The primary keratinocytes demonstrated a stimulatory effect on the basal expression of CD markers in THP-1 cells. CD54 expression in the medium increased to 95.2%. Given that no significant increase was possible upon stimulation, only CD86 expression was determined. No stimulation of CD86 expression was observed in the presence of NPs or negative controls. However, a stimulation effect was evident in the positive control, DNCB (Table 4).

**Figure 7:**
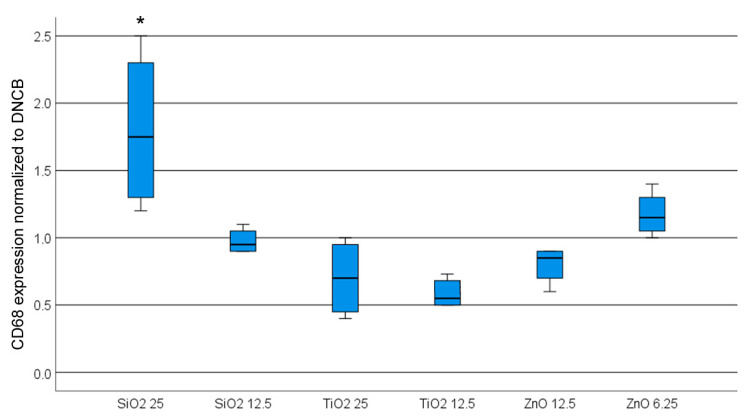
CD86 expression of THP-1 cells in the COCAT_primary keratinocyte when primary keratinocytes were exposed to NPs prior to contact with the sensitizer (positive control) 2,4-dinitrochlorobenzene (DNCB). Significant changes are indicated by an asterisk (*p* < 0.05).

The pre-incubation of primary keratinocytes with 25 µg/mL SiO_2_ resulted in a notable enhancement in the response of THP-1 cells to DNCB, as illustrated in Figure 7. The remaining NPs did not significantly impact the THP-1 cell response to DNCB.

### 3.7. COCAT_Transwell Assay

When DNCB was introduced to the apical compartment, solely to expose the keratinocytes, no discernible increase in CD54 and CD86 expression was observed in the THP-1 cells. In this experimental set-up, the HaCaT cells can only communicate with the THP-1 cells via cytokines. Upon addition of the chemicals to the basal compartment, an increase in CD expression was observed, however, the response remained lower than that observed in direct co-culture (COCAT). The CD54 expression level increased to 126 ± 2 in comparison to 165 ± 5, while the CD86 expression level increased to 1438 ± 1192 in comparison to 3452 ± 1252 in the COCAT assay (Table 5).

**Figure 8:**
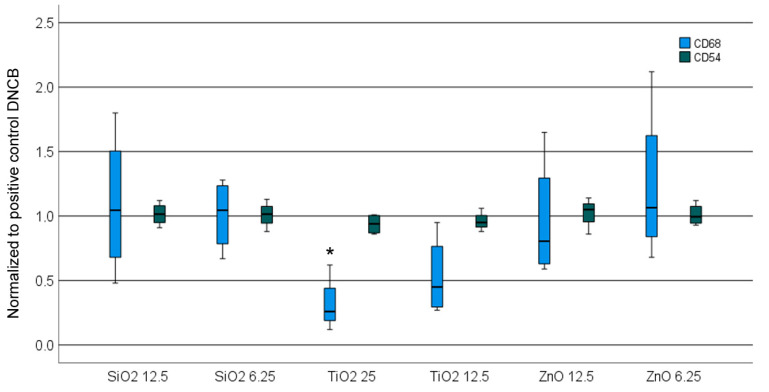
CD86 and CD54 expression of THP-1 cells after exposure to NPs in the COCAT_transwell assay followed by sensitizer 2,4-dinitrochlorobenzene (DNCB). Significant changes are indicated by an asterisk (*p* < 0.05).

The pre-incubation with SiO_2_ resulted in a slight elevation of CD86 expression relative to DNCB exposure alone, as illustrated in Figure 8. The remaining NPs did not exert an increased response to DNCB in THP-1 cells.

## 4. Discussion

The objective of this study was to investigate the activation of keratinocytes and DCs by exposure to SiO_2_, TiO_2_, and ZnO NPs in mono- and co-culture. This is a significant point, as keratinocytes and DCs are essential and fulfill different roles in the sensitization process. Direct contact between keratinocytes and Langerhans cells (LCs), the DCs of the skin, occurs in the human skin and LCs may counteract the pro-inflammatory activity of surrounding keratinocytes [35]. Due to the interaction, results obtained by direct co-culture should be more representative of the human situation. The results obtained differed depending on the culture method employed, and confirmed the importance of direct contact of the different cell types.

In the case of keratinocyte activation alone, the method proposed by Jeon et al. (2019) was employed [33], however, the results were not interpretable due to the low levels of interleukin secretion observed in the HaCaT cells. The assay is seldom employed for the classification of sensitizers, which may be attributed to the lack of assay specificity, as IL-1α is also released upon irritation [36].

The U-SENS™, which is an accepted in vitro test for sensitization, was employed to assess separately DC activation [29]. As documented in the literature, exposure to 10–1430 nm SiO_2_ particles has been shown to increase CD86 expression in THP-1 cells [37]. Similarly, 295 nm TiO_2_ NPs have been demonstrated to elevate CD86 expression in U937 cells [38]. Additionally, 30–40 nm ZnO NPs have been observed to enhance CD54 and CD86 expression in THP-1 cells [11]. In contrast to the aforementioned reports, no activation was observed in the presence of the NPs alone in our study. It is plausible that the disparate outcomes pertaining to DC activation can be attributed to the fact that the other studies employed considerably higher concentrations of NPs to achieve activation, specifically 0.6 mg/mL for SiO_2_, 50–200 µg/mL for TiO_2_, and 25.8 µg/mL for ZnO NPs. Furthermore, the selection of the CD activation assay influenced the results because a discrepancy was observed in 18% of compounds between U-SENS™ and h-CLAT, both of which assess DC activation [39]. In combination with a sensitizer, TiO_2_ was observed to decrease CD86 expression, whereas ZnO was found to increase it (Table 6). No THP-1 or U937 cell data were identified in the literature for comparison.

In contrast with the approach proposed by Lee et al. [39], which involved combining separate assays for the classification of sensitizers, we employed a keratinocyte/THP-1 co-culture (COCAT) system. Three versions of the COCAT system were employed for the purpose of evaluating the interaction between DCs and keratinocytes. LCs, which are located in the basal and suprabasal layers of the epidermis in an activated state, are capable of taking up antigens outside the barrier [18]. This scenario was replicated through the co-culture of keratinocytes and THP-1 cells within the same compartment.

Our findings revealed that the requisite elevation in CD expression within this co-culture system was exclusive to THP-1 cells within a specific passage range. The tendency of continuous cell lines to exhibit genetic instability may result in the emergence of genetically distinct subtypes within a given cell line. It is essential to consider this aspect in the experimental design and to include it in the published work, as there is a well-established discrepancy in the outcomes between high and low passage numbers [40,41]. The indication of the passage number is crucial, despite the fact that the number itself may lack significant meaning. This is because the supplier often neglects or is not able to indicate this information at the time of shipment, and further counting by different laboratories may yield disparate results.

As documented by other research groups, the presence of keratinocytes enhanced the expression of CD86 and CD54 by THP-1 cells [42]. Furthermore, the response to DNCB was observed to be higher in the co-cultures of our study. It has been documented that the enhancement in sensitivity is especially pronounced for substances where the involvement of keratinocytes may be crucial for the non-enzymatic oxidation of prehaptens or the metabolic transformation of prohaptens [43]. As expected, the co-culture method increased the specificity of the assay in our study. This phenomenon is explained by the fact that increased CD54 expression in THP-1 cells can be induced by irritants that do not induce activation in the presence of HaCaT cells [44]. The results of the present study demonstrate that SiO_2_ alone (Table 3) and in combination with TCA increased CD86 expression (Figure 6). To the best of our knowledge, no studies have evaluated nanoparticles in such a co-culture system. The increase in sensitization by SiO_2_ may have clinical relevance due to the antibacterial properties of mesoporous silica particles loaded with cinnamaldehyde and ZnO integrated into hydroxyethyl cellulose-based composite films. These composite films may find application as wound dressings [45]. Since Hirai et al. [15] showed less exacerbation of atopic dermatitis after administration of 300–1000 nm than 30–100 nm SiO_2_, weaker effects of the larger particles are likely. Conclusions about the importance of NP size in sensitizing potential are complicated by the fact that SiO_2_ NPs of similar size (~20 nm) showed no effect in one in vivo study but showed an indication of sensitization in another [12,13].

In an alternative version of COCAT, primary keratinocytes extracted from the foreskin are cultivated on a transwell and THP-1 cells in the lower chamber [44]. It has been documented that the presence of primary keratinocytes may elevate CD54 expression by THP-1 cells to exceedingly high levels, rendering it unfeasible to discern stimulation by sensitizers [46]. As this was also the case in our study, we limited our evaluation to CD86 expression. With the exception of the lack of response to SiO_2_ NPs alone, the response in COCAT_primary keratinocytes was comparable to that observed in COCAT (Table 6). This system appears to exhibit reduced sensitivity to the activating effects of NPs when tested alone. Furthermore, the combination of primary keratinocytes and monocyte-derived DCs introduces significant inter-donor variability, which is a limitation of this system [47].

In the third iteration of the COCAT system, HaCaT cells were cultured on membranes, resulting in the formation of a stratified layer of keratinocytes. In this system, exposure to TiO_2_ NPs resulted in a notable reduction in the response to DNCB, as evidenced in Table 6. The response was comparable to that observed in U-SENS™, which may be attributed to the fact that direct contact between HaCaT cells and THP-1 cells was prevented by the Transwell membrane.

The effects of NPs and sensitizers on mono- and co-cultures of Ha-CaT and THP-1 cells yielded disparate outcomes. In a comparable analysis of co- and mono-cultures of HaCaT and THP-1 cells, the sensitizers cobalt chloride, methylisothiazoline, and p-phenylenediamine elicited disparate patterns of cytokine secretion [48]. With the exception of a few instances, cytokine secretion was observed to be lower in the co-cultures than in any of the monocultures.

One limitation of this study is that the combination of nanoparticles with only two sensitizers was tested, and the evaluation was limited to a single time point. Furthermore, ultraviolet radiation was not employed in this study, despite the fact that nanoparticles are utilized in sun protection and UV-B is recognized to facilitate the increased dissociation of Zn^2+^, leading to the accumulation of Zn^2+^ and the induction of oxidative stress and inflammation [49].

The findings of our study underscore the significance of direct interaction between keratinocytes and DCs. The findings demonstrated that metal oxide NPs exert a distinct influence on the response to sensitizers. The present study did not reveal any size-dependent effects of the NPs, which ranged in size from 5 to 90 nm. Similar results were previously published by Wareing et al. for SiO_2_, TiO_2_, and ZnO NPs with primary sizes of 5–190 nm [28]. In vivo, the aggravation of atopic dermatitis was more pronounced after the administration of 30–100 nm than of 300–1000 nm SiO_2_ NPs, indicating a size-dependent effect [15]. This result may appear surprising, given that the agglomeration of metal oxide NPs in media is a common phenomenon (e.g., [50]) and has also been observed in this study.

## 5. Conclusions

Similar to the results of sensitization studies conducted on different in vivo systems, the response to NPs varied between cellular models. The results obtained from co-cultures with keratinocytes in direct contact differed from those observed in monocultures of U937 cells. However, co-cultures with either primary keratinocytes or HaCaT cells exhibited a similar response. When the monocytes were separated from the keratinocytes by a membrane, the response of the co-culture system was found to be more similar to that of U-SENS™. The comparison of COCAT with COCAT_transwell indicates that direct contact between keratinocytes and DCs is a crucial factor in the action of sensitizers. Upon direct contact with keratinocytes, the modulating effect of TiO_2_ and ZnO NPs on the sensitizers decreased, and that of SiO_2_ increased. However, it is challenging to align these findings with those from in vivo studies due to the inconsistency in reported outcomes in animal models and the scarcity of data comparing the impact of NPs alone and in combination with a sensitizer.

The translation of in vitro test results into the risk of human sensitization is a challenging endeavor, as numerous parameters exert influence over this phenomenon. These include product composition, frequency of application, duration of exposure, condition of the skin site, and various inter-individual parameters, particularly genetics, pre-existing disease state, and sensitive subpopulations [51]. The need to replace animal studies requires the development of (more) representative in vitro models. The most promising appears to be reconstructed human epidermis with integrated LCs, termed RhE-LC [52]. This model was developed by L’Oréal and is marketed as SkinEthic™. However, the major limitations of this model were the absence of the dermal compartment, and, therefore, the absence of LC migration, and the logistical complexity of constructing the model due to the reliance on cultured primary keratinocytes and cord blood-derived LC.

## Figures and Tables

**Table 1:** Hydrodynamic sizes (Z-Ave), polydispersity index (PdI), and zeta potential (ZP) of the particles dispersed in DMEM or RPMI medium with 10% FBS prior to the exposure and after 24 h.

	Time (h)	Z-Ave (nm)		PdI		ZP (mV)	
**Particle**		DMEM	RPMI	DMEM	RPMI	DMEM	RPMI
**14–23 nm SiO_2_**	0	723 ± 110	376 ± 70	0.68 ± 0.14	0.39 ± 0.01	0.6 ± 1.8	13.6 ± 1.4
	24	127 ± 7	142 ± 10	0.33 ± 0.01	0.38 ± 0.06	13.9 ± 0.8	14.2 ± 1.1
**5–6 nm TiO_2_**	0	690 ± 23	766 ± 86	0.71 ± 0.02	0.45 ± 0.07	12.4 ± 1.4	11.4 ± 1.2
	24	478 ± 75	365 ± 15	0.53 ± 0.12	0.50 ± 0.03	14.5 ± 0.9	14.5 ± 1.2
**70–90 nm ZnO**	0	232 ± 147	283 ± 44	0.68 ± 0.25	0.62 ± 0.09	12.3 ± 1.3	12.8 ± 0.7
	24	143 ± 18	187 ± 10	0.30 ± 0.03	0.38 ± 0.03	15.5 ± 1.4	15.2 ± 0.5

**Table 2:** Inductions of CD86 expression in U937 cells by chemicals and NPs alone.

Stimulant	Concentration (µg/mL)	S.I. (Mean ± SD)
Lactic acid (LA)	50	103 ± 22
2,4,6,-Trinitro-benzene-sulfonic acid (TNBS)	25	835 ± 202
paraformaldehyde (PFA)	0.2	361 ± 206
trans cinnamaldehyde (TCA)	1.0	498 ± 137
2,4-dinitrochlorobenzene (DNCB)	0.5	211 ± 56
Silica (SiO_2_)	12.5	121 ± 12
Titanium dioxide (TiO_2_)	12.5	74 ± 12
Zinc oxide (ZnO)	6.25	117 ± 50

**Table 3:** Induction of CD54 and CD86 expression in THP-1 cells in co-culture with HaCaT by chemicals and NPs alone.

Stimulant	Concentration (µg/mL)	CD54, S.I. (Mean ± SD)	CD86, S.I. (Mean ± SD)
Sodium dodecyl sulfate (SDS)	41.5	98 ± 8	111 ± 18
Trans cinnamaldehyde (TCA)	7.5	139 ± 43	172 ± 73
2,4-dinitrochlorobenzene (DNCB)	3	192 ± 63	3427 ± 514
Silica (SiO_2_)	12.5	123 ± 5	1109 ± 187
Titanium dioxide (TiO_2_)	12.5	98 ± 2	171 ± 11
Zinc oxide (ZnO)	12.5	85 ± 10	154 ± 77

**Table 4:** Induction of CD86 expression in THP-1 cells in co-culture with primary keratinocytes by chemicals and NPs alone.

Stimulant	Concentration (µg/mL)	S.I. (Mean ± SD)
Sodium dodecyl sulfate (SDS)	2.8	104 ± 24
2,4-dinitrochlorobenzene (DNCB)	0.5	607 ± 68
Silica (SiO_2_)	12.5	86 ± 10
Titanium dioxide (TiO_2_)	12.5	51 ± 11
Zinc oxide (ZnO)	12.5	78 ± 10

**Table 5:** Induction of CD54 and CD86 expression of THP-1 cells in COCAT_transwell assay by chemicals and NPs alone.

Stimulant	Concentration (µg/mL)	CD54, S.I. (Mean ± SD)	CD86, S.I. (Mean ± SD)
Sodium dodecyl sulfate (SDS)	2.8	89 ± 7	93 ± 26
2,4-dinitrochlorobenzene (DNCB)	0.8	126 ± 21	1438 ± 1192
Silica (SiO_2_)	12.5	91 ± 8	121 ± 5
Titanium dioxide (TiO_2_)	12.5	91 ± 3	63 ± 2
Zinc oxide (ZnO)	12.5	95 ± 2	94 ± 4

**Table 6:** Overview of results after exposure to the NPs alone, and after subsequent exposure to sensitizer (+).

Modell	SiO_2_	TiO_2_	ZnO
U-SENS™		+: Decrease	+: Increase
COCAT	−: Increase +: Increase		
COCAT_primary keratinocytes	+: Increase		
COCAT_transwell		+: Decrease	

## Data Availability

Data are available from the authors.

## Supplementary Materials

The following supporting information can be downloaded at: https://www.mdpi.com/article/10.3390/nano14221811/s1, Table S1: Additional particle information. Figure S1: Additional cytotoxicity data Refs. [53,54,55] are cited in the Supplementary Materials.
