# Supplementary material for: Skin Sensitization Potential of Sensitizers in the Presence of Metal Oxide Nanoparticles In Vitro
Source: Nanomaterials (Basel). 2024 Nov 12;14(22):1811. doi: 10.3390/nano14221811 (PMC11597535; doi:10.3390/nano14221811)
Supplement: Supplementary file 1 [file nanomaterials-14-01811-s001.zip › nanomaterials-3277219-supplementary update.pdf]

Skin sensitization potential of sensitizers in the presence of metal oxide nanoparticles in vitro

Claudia Meindl [1], Kristin Öhlinger [1], Verena Zrim [1], Jennifer Ober[1], Ramona Jeitler [2], Eva Roblegg [2], and Eleonore Fröhlich [1,*]

[1] Center for Medical Research, Medical University of Graz, Stiftingtalstr. 24, 8010 Graz, Austria; claudia.meindl@medunigraz.at; kristin.oehlinger@medunigraz.at; verena.zrim@medunigraz.at, Jennifer.ober@medunigraz.at

[2] Institute of Pharmaceutical Sciences, Department of Pharmaceutical Technology, Karl-Franzens-University of Graz, Humboldtstr, 46, 8010 Graz, Austria; ramona.jeitler@uni-graz.at; eva.roblegg@uni-graz.at

* Correspondence: Eleonore.froehlich@medunigraz.at; Tel.: +43 31638573011

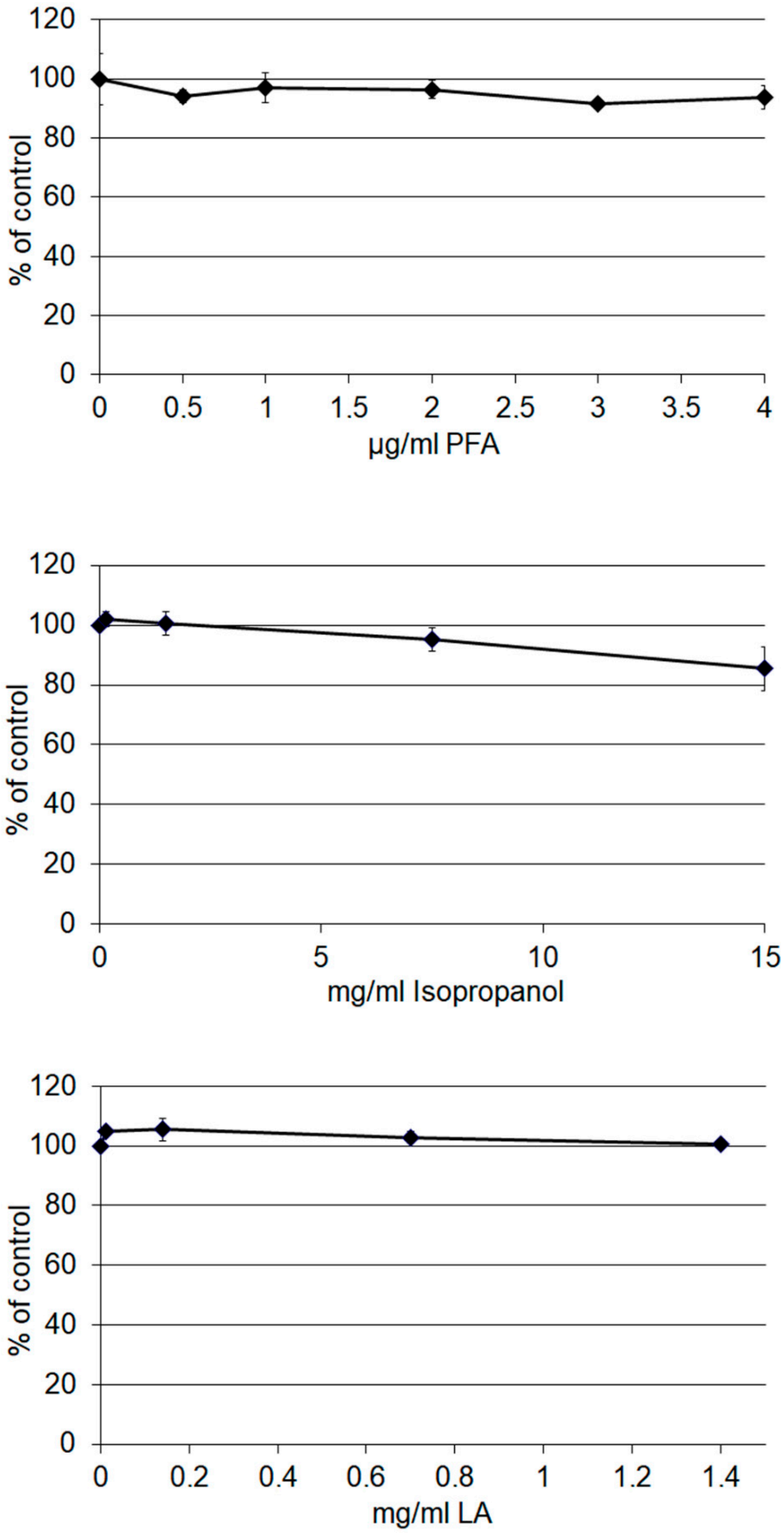

Figure S1. Cell viability is indicated as % of (medium or solvent) control. Viability was determined for paraformaldehyde (PFA), isopropanol, and lactic acid (LA) in HaCaT keratinocytes.

Table S1: Information provided by JRC (https://joint-research-centre.ec.europa.eu/system/files/2016-06/JRC%2520Nanomaterials%2520Repository-List%2520of%2520Representative%2520Nanomaterials-201606.pdf)

**JRC ID1**	**Former NM code**	**Type of material**	**Primary particle size [nm]**	**Particle size distribution [nm]**	**Specific surface area [m² /g]**	**Other information**	**Reference**
JRCNM02000a	NM-200	Silicon dioxide	14-23	<100: 89%<br><50: 70%<br><10: 2%	204	Synthetic amorphous silica, precipitated	[1]
JRCNM62101a	NM-110	Zinc oxide	70-90	N/A	N/A	Uncoated	[2]
JRCNM01001a	NM-101	Titanium Dioxide	5-6	<100: 95%<br><50: 77%<br><10: 11%	170/316	Anatase	[3]

References

1. Rasmussen, K.; Mech, A.; Mast, J.; De Temmerman, P.; Waegeneers, N.; Van Steen, F.; Pizzolon, J.; De Temmerman, L.; Van Doren, E.; Jensen, K., et al. *Synthetic Amorphous Silicon Dioxide (NM-200, NM-201, NM-202, NM-203, NM-204): Characterisation and PhysicoChemical Properties*; Publications Office of the European Union: Luxembourg, 2013; doi:10.2788/57989.
2. Singh, C.; Friedrichs, S.; Levin, M.; Birkedal, R.; Jensen, K.; Pojana, G.; Wohlleben, W.; Schulte, S.; Wiench, K.; Turney, T., et al. *NM-Series of Representative Manufactured Nanomaterials - Zinc Oxide NM-110, NM-111, NM-112, NM-113: Characterisation and Test Item Preparation*; Publications Office of the European Union: Luxembourg, 2011; doi:10.2787/55008
3. Rasmussen, K.; Mast, J.; De Temmerman, P.; Verleysen, E.; Waegeneers, N.; Van Steen, F.; Pizzolon, J.; De Temmerman, L.; Van Doren, E.; Jensen, K., et al. *Titanium Dioxide, NM-100, NM-101, NM-102, NM-103, NM-104, NM-105: Characterisation and Physico-Chemical Properties*; Publications Office of the European Union: Luxembourg, 2014; doi:10.2788/79554.
